# The Bridle Mark System on Bottlenose Dolphins (*Tursiops truncatus*): Pigmented Facial Features Supplement Photo-Identification

**DOI:** 10.3390/ani16121857

**Published:** 2026-06-16

**Authors:** Barbara J. Brunnick, Graysen D. Boehning, Stefan Harzen

**Affiliations:** Taras Oceanographic Foundation, West Palm Beach, FL 33407, USAharzen@taras.org (S.H.)

**Keywords:** marine mammal, dolphin, *Tursiops*, identification, bridle mark, facial feature

## Abstract

Research on free-ranging animal species, which extends from the breadth of stock identification and population dynamics to fine-scaled analysis of behavior, depends on identifying individuals. Recognizing wild bottlenose dolphins (*Tursiops truncatus*) primarily depends on photographs of distinct patterns of notches which eventually develop on the trailing edge of most dorsal fins. Although photo-identification has advanced to include computer-aided fin identification software, there can still be error related to dolphins whose identifying patterns are subtle, obscured, or absent (“clean fins”). Facial pigment patterns on dolphin species were described as bridle marks over a hundred years ago. The purpose of this descriptive study was to evaluate bridle mark presence, distinctiveness, and permanence as a supplement to dorsal fin photo-identification. A 17-year population census of 860 bottlenose dolphins in Palm Beach, Florida, USA, includes 458 dolphins with photographic records showing bridle marks. A subset of 30 dolphins, based on an encounter history spanning at least 7 years, was coded using a 17-point system. The results confirmed bottlenose dolphin bridle mark distinctiveness and permanence on all 30 dolphins. In conclusion, bridle marks can supplement photo-identification as a double-mark system to improve accuracy. This tool is helpful for recognizing calves with clean dorsal fins post weaning and adults without dorsal fin markings.

## Simple Summary

It is important to identify individual cetaceans (porpoises, dolphins, and whales) to study their populations, time budgets, habitat use, and social behavior. Researchers primarily use photo-identification to identify individual bottlenose dolphins from unique patterns of rips and tears that develop along the trailing edge of the dorsal fins. Whereas most dolphins develop identifying marks by adulthood, there are situations where supplemental data could help avoid mistaken identity. One is subsequent damage to recorded identifiable fin patterns. The other is calves, which are identified by their association with their assumed mothers until they acquire identifying dorsal fin marks. Should these “clean” finned calves become independent from their mothers before obtaining identifiable marks, it is difficult, if not impossible, to recognize them, thus introducing error into the database. Dolphin bridle marks are naturally occurring, distinctive, permanent, and stable facial features that can be photographically monitored to verify individual identities. These marks are proposed as a useful supplement for current photo-identification methods to reduce error.

## 1. Introduction

Marine mammals host a variety of physical features that researchers use to identify individuals in the wild. The extensive use of natural markings for identification of individual cetaceans was pioneered by Gunter (1942) [1] and Caldwell (1955) [2], allowing studies to originally focus on distribution, migration, and general life history (Schevill and Backus, 1960; Caldwell and Golley, 1965) [3,4]. The onset of a few committed long-term study programs made it clear that individual discernment could also provide information on abundance, survivorship, reproduction, kinship, behavior, and the distinction of discrete populations, which are all important information for the management of a top predator over time (Katona and Whitehead, 1981; Norris et al. 1985; Wells et al. 1987; Scott et al. 1990; Wells and Scott, 1990) [5,6,7,8,9].

The delphinid dorsal fin is primarily composed of cartilage, tapering in thickness toward the trailing edge, which is prone to nicks and notches obtained through injury or interaction with conspecifics and potentially hetero specifics. The trailing edge tissues do not regenerate, making them an ideal area for identification (ID) marks (Wells, 2018) [10].

Identification of dorsal fin irregularities was originally introduced for two species of delphinids: killer whales (*Orcinus orca*) (Bigg et al. 1976; Balcomb et al. 1982; Dahlheim, 1997) [11,12,13] and bottlenose dolphins (*Tursiops truncatus*) (Würsig and Würsig, 1977; Shane, 1977) [14,15]. Since then, the physical feature for the identification of both *Orcinus* and *Tursiops* was the trailing edge of the dorsal fin; however, killer whale identification is further bolstered by the inclusion of the saddle patch behind the dorsal fin as a second identifier (Balcomb et al. 1982; Evans et al. 1982; Scott et al. 1990) [8,12,16].

The distinctiveness and permanence of natural marks made them suitable for population size estimation through mark recapture methods (Hammond, 1986; Cockcroft et al. 1992; Agler, 1992; Rosel et al. 2011) [17,18,19,20]. The identification process evolved as new technology emerged and methodologies improved. The focus remained on the dorsal fin of the bottlenose dolphin even as limitations were discovered (Urian et al. 2015) [21].

### 1.1. Photo-Identification

The process of photo-identification became the standard means to document the identity of individual wild delphinids (Defran et al. 1990) [22]. The use of digital photographs to capture and collect dorsal fin images over time revolutionized photo-identification (Mazzoil et al. 2004) [23]. By the early 1990s, computerized photo-ID collections were used by long-term programs of both odontocetes and mysticetes worldwide, making data sharing between research programs possible (Hammond et al. 1990; Urian and Wells, 1996; Tyson-Moore et al. 2022; Pirotta et al. 2026) [24,25,26,27].

Natural marks can be variable, and the success of photo-identification is subject to limitations. Whereas notches in the trailing edge of dolphin dorsal fins are permanent, their appearance may be altered. Norris et al. 1985 [6] documented changes in these markings over a 1–2-year period. Bigg 1982 [28] found that marks can elongate and become shallow over time. Therefore, not all individuals can be re-identified over long sampling intervals and change can introduce several inadvertent sources of error (Hammond, 1986) [17]. Once the documented dorsal fin identifier of a bottlenose dolphin is obscured or damaged over time, this animal can be mis-identified or lost from the census (Bichell et al. 2018) [29]. The quality of ID photographs and the distinctiveness of identifiers also impact the success of photo-identification of cetaceans (Urian et al. 2015; Friday et al. 2000; Ronje et al. 2020; Tyson-Moore et al. 2022) [21,26,30,31]. Some errors can be avoided by using only high-quality photographs in the identification process (Urian et al. 2015; Friday et al. 2000; Stevick et al. 2001; Ronje et al. 2020; Tyson-Moore et al. 2022) [21,26,30,31,32].

Natural identifiable marks are not equally distributed throughout the population. Individuals with highly distinguishable marks are accurately identifiable even in the poorest photograph or from the boat. Those with less discernable marks are difficult or impossible to identify, leading to potential error in datasets (Hammond, 1986; Urian et al. 2015; Stevick et al. 2001; Tyson-Moore et al. 2022) [17,21,26,32].

### 1.2. Clean Fins

In all studies of free-ranging bottlenose dolphins, those which lack identifying marks, whether they are dorsal fin patterns or body scars, comprise the segment of the population with the highest potential for being overlooked or inaccurately documented in the censuses (Machernis et al. 2021; Tyson-Moore et al. 2022) [26,33]. They are called “clean fins” and cannot be recognized from one encounter to the next. A large proportion of dolphins in the clean fin category are calves which are weaned and have reached a level of independence from their mothers before obtaining suitable identifying marks (Urian et al. 2015) [21].

Relatedness is an important factor in population dynamics, making the identity of cow/calf pairs imperative. Determining how siblings or cousins interact in a society can explain how behaviors and social structure may benefit the reproductive success of the individual and species (Trivers, 1985; Mann and Smuts, 1999; Gibson and Mann, 2008; Greenfield et al. 2022) [34,35,36,37]. Therefore, finding a means to confidently monitor individual calves through adolescence and beyond is important (Connor and Norris, 1982; Diaz-Aguirre et al. 2020) [38,39].

### 1.3. A Second Mark System

When natural identifying marks are absent or the longevity of natural marks is not guaranteed, it is advantageous to use two or more independent marks on the same individual (Scott et al. 1989; Aubin et al. 2000; Yamamoto et al. 2022) [40,41,42]. Some field researchers have routinely used a dual mark process (both dorsal marking and callosity patterns) to verify individuals in populations of right whales (*Eubalaena* spp.) (Payne et al. 1983; Kraus et al. 1986; Bannister, 1990) [43,44,45]. Humpback whales (*Megaptera novaeangliae*) are generally identified by marks on the tail flukes. However, several research programs have been looking for another identifier and collect photos of the dorsal fin and/or pectoral fins, which are helpful when whales do not fluke-up when diving (Katona et al. 1979; Barlow et al. 2011; Franklin et al. 2020) [46,47,48]. More recently, top-down images have been incorporated into an aerial-ID catalog for humpback whales to be used with drone imagery (Evans et al. 2026) [49].

A second mark system would also be a helpful tool in following cow/calf behavior of delphinids through weaning (Brunnick and Herzing, 1998; Brunnick, 2000; Bichell et al. 2018) [29,50,51,52,53,54,55,56,57] and help track individual development over long periods of time (Brunnick and Herzing, 1998; Mann and Smuts, 1999; Brunnick, 2000; Greenfield et al. 2022) [35,37,50,51].

### 1.4. Bridle Marks

A largely overlooked second mark system emanates from bridle marks on dolphin faces, mostly composed of hyperpigmented stripes extending from the eye and the blowhole to the apex of the melon (Perrin, 1997) [52]. Bridle marks have long been documented in genera of small cetaceans, including *Stenella* and *Tursiops* (Guldberg and Nansen, 1894; Perrin, 1969; Mitchell, 1970; Perrin, 1997; Brunnick and Herzing, 1998; Brunnick, 2000) [50,51,52,53,54,55].

In a late-19th century study of collected fetal tissues, Guldberg and Nansen (1894) [53] first defined the line extending across the back of the head in *Lagenorhynchus* spp. and *Orcinus* spp., as the “Anlage of the Bridle”. They did not refer to the lines to the eye or blowhole features, which appear later in fetal development (Perrin, 1997) [52]. The term “bridle” was next used by Mitchell (1970) [55] to describe the stripes that extend from the eye and the blowhole to the apex of the melon of delphinids.

Perrin suggested variation in the coloration of *Stenella* spp. was striking enough to be significant, possibly related to social structure. He found “between-school differences” in the coloration of what he called the “flipper band and the cape” (later identified as parts of the bridle mark system) of tropical *Stenella*, and suggested differences may inform conspecifics of group membership during periods when schools gathered for feeding or breeding purposes (Perrin, 1969) [54].

Later, Perrin determined that bridle mark systems were present in the families of Delphinidae and Phocoenidae but not Monodontidae, and he described these marks in some detail through fetal development using tissues from dolphins stranded or killed by tuna fisheries. In an exhaustive study, Perrin found the bridle mark system in all 32 delphinoid species, including *Tursiops*, and further suggested the eye patch without a stripe may be primitive as it occurs in non-delphinoid cetaceans (Perrin, 1997) [52].

Brunnick and Herzing found bridle marks were present and helpful in the identification of *Stenella frontalis* calves prior to weaning and onward through adolescence. They reported that although bridle marks can be difficult to discern on newborns, they do become more distinct shortly after birth. Because spotted dolphin calves are born without identifying spots and can wean before developing them, bridle marks proved to be a valuable alternative for tracking young spotted dolphins during that early phase of their lives (Brunnick and Herzing, 1998) [50].

Genov et al. 2017 [56], studying bottlenose dolphins in the Gulf of Trieste, Adriatic Sea, Italy, suggested dolphin faces may be symmetrical (left to right) and distinctive enough for humans to distinguish individual dolphins (Genov et al. 2017) [56]. However, there was no reference to any specific facial feature. Spencer (2018) [57] found that variations in facial features of dolphins are discernable by humans who have experience in photo-identification. Both studies found matching facial features were significantly different to what would be expected from random matching; however, neither study used the term bridle marks (Genov et al. 2017; Spencer, 2018) [56,57].

The main purpose of this analysis is to confirm three assumptions:

(1) *Tursiops* do in fact have visible bridle marks, as described by Perrin (1997) [52].
(2) *Tursiops* bridle marks are highly variable across a population but distinct on individuals.
(3) *Tursiops* bridle marks remain stable over time.

If all three assumptions are true, bridle marks would serve as a reliable supplemental identifier for the bottlenose dolphin when dorsal fin marks are in transition or obscured by additional damage, and especially for “clean fins”, notably in dolphin calves.

## 2. Materials and Methods

### 2.1. Study Area

**Figure 1:**
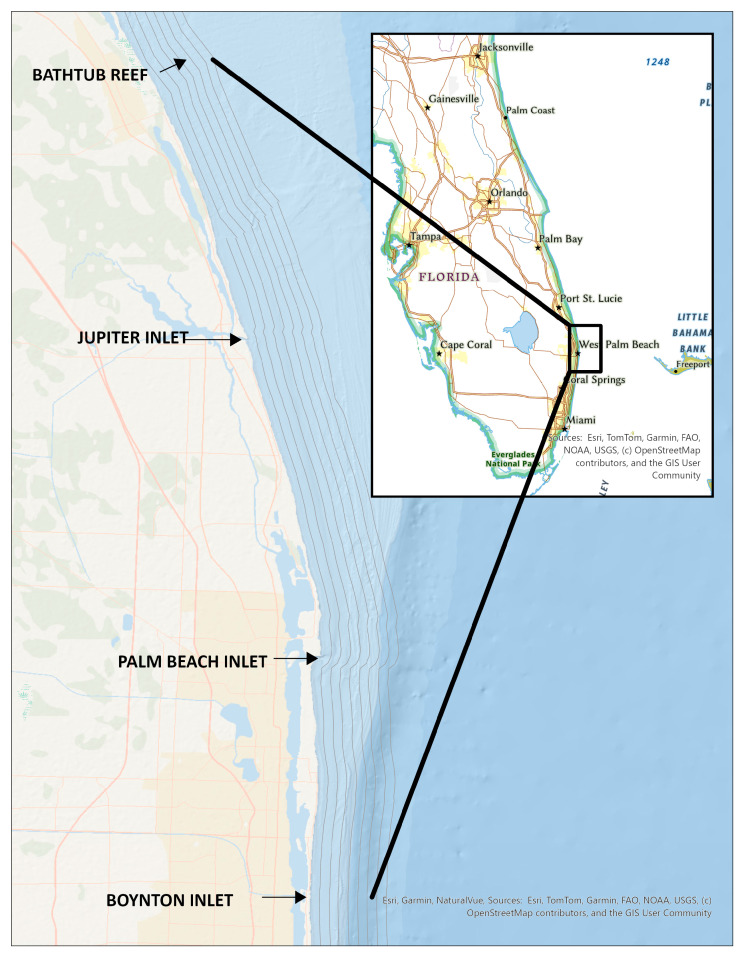
The study site in Southeast Florida, USA. Lines running parallel to the shore mark 0.5 km distances.

The study area comprises coastal Palm Beach and Martin Counties from Bathtub Reef (27°11.017 N, 80°0.017 W) in the north, to Boynton Beach Inlet (26°54.017 N, 80°4.017 W) in the south. This study site is characterized by coastal, open ocean without natural barriers such as banks, bays, and estuaries (Figure 1). The underwater habitat is predominately sandy bottom sloping to the east toward a reefal complex system located approximately 1.6 km from shore, where the depth exceeds 25 m.

### 2.2. Study Period

Data were collected between 2008 and 2024 under NOAA General Authority permits (#s 18152, 13386, and 22291). Highly variable weather conditions in the open shore environment ultimately dictated the time, frequency, direction, and duration of our surveys.

### 2.3. Survey Design

The photo-identification data used in this study were collected during regular boat surveys conducted throughout the study area. To facilitate data collection, the area was divided into two transects: one from Jupiter Inlet south to the Palm Beach Inlet (21 km) and the other from Palm Beach Inlet to the Boynton Inlet (27 km). Surveys were designed to run approximately 1.2 km from, and parallel, to the coast.

Our research platform was a ridged hull inflatable 34’ center console vessel with two outboard gas engines. During each survey, the research vessel proceeded along a pre-designated survey route, typically at a speed of 5 to 10 knots.

The survey crew, consisting of an experienced skipper, the lead researcher, and one or more onboard observer(s), kept a consistent visual search in all directions around the boat, using 7 × 50 waterproof Bushnell binoculars.

Encounters began when dolphins were detected during a survey. At this point, a GIS waypoint was collected marking the time and location. The vessel was then maneuvered close enough to a group of dolphins to acquire suitable photographs of the dorsal fins. Extreme caution and consideration for the well-being of the dolphins dictated the speed and degree of approach, following some norms, such as always approaching from behind and to one side, at a speed to match the dolphins during travel.

During each encounter, photographers endeavored to photograph every individual dolphin possible within a visible range. At the end of each encounter, we returned to the transect and resumed the survey in search of additional dolphins in the study area.

### 2.4. Field Study Effort

Between 2008 and 2024, a total of 653 surveys resulted in 370 encounters with bottlenose dolphins. Survey duration averaged two hours (M = 122 min, SD 56.85, Range = 15–302 min). Survey duration was longer when dolphins were encountered. The duration of encounters with dolphins averaged about an hour (M = 50 min, SD 37.59, Range = 2–233 min).

### 2.5. Photographic Equipment and Protocol

Photographic data were collected with Nikon SLR cameras (D70, D200, D300, and D500), equipped with various telephoto lenses (80–200 mm, 80–400 mm).

Photographs of dorsal fins were taken when the dolphin surfaced to breathe and used to identify individuals following the standards developed for dolphin photo-ID studies (Hammond, 1986; Hammond et al. 1990; Urian and Wells, 1996; Mazzoil et al. 2004; Boehning et al. 2023) [17,23,24,25,58].

### 2.6. Image Quality and Selection Criteria

Only photographs in which the full dorsal fin is visible, in focus, and orientated at an approximate 90-degree angle were used for identification and included in the ID catalog. Furthermore, a digital photo database of all identifiable individuals was accumulated and maintained to include images of the heads, flukes, and other body parts.

To evaluate bridle marks for presence, distinctiveness, and permanence, the extensive photo database was searched for images of individuals showing their head area, which were then examined for the presence of bridle marks.

### 2.7. Sample Selection

**Figure 2:**
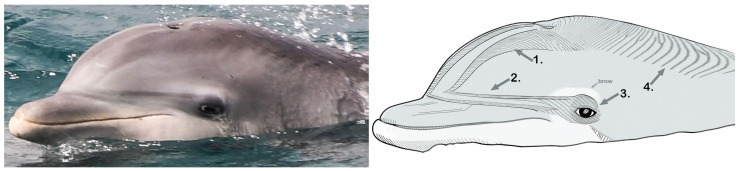
Side view of the common bottlenose dolphin bridle mark: (**1**) melon line; (**2**) eye line; (**3**) eye patch; (**4**) pre cape. Photographic image collected under NOAA GA# 18152. © Harzen/Brunnick. 2018.

The 15-year census of bottlenose dolphins in our study comprised more than 860 dolphins, 458 (53%) of which had usable photos of the head (Figure 2) that were examined for the presence of a bridle mark system. Dolphins recorded in more than one year (*n* = 122) had more extensive photo records associated with higher site fidelity (Boehning et al. 2023) [58]. In order to assess distinctiveness and permanence—i.e., whether bridle marks are inconsistent between individuals, and stable over time—we selected the individuals for which we had a photo record spanning at least 7 years, with the exception of one calf whose record spanned from birth year through year 3. All animals chosen had a photo record consisting of both fully visible bridle marks and dorsal fin patterns for positive identification. A final count of *n* = 30 dolphins was then coded and evaluated for distinctiveness within this study.

### 2.8. Bridle Mark Coding System

Photo records of these 30 dolphins were assigned a qualitative code based on descriptive qualities of four facial features defined by Perrin 1997 [52]. The authors examined and independently coded each bridle mark, resulting in an identical sequence of numbers for each animal that was converted into a 17-digit code assigned to each dolphin.

**Figure 3:**
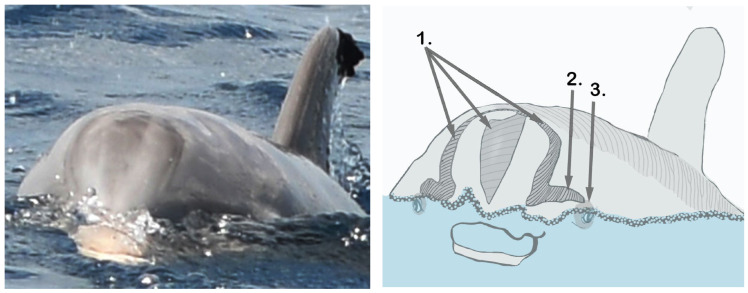
Front view of the common bottlenose dolphin bridle mark: (**1**) melon line; (**2**) eye line; (**3**) eye patch. Photographic image collected under NOAA GA# 22291. © Harzen/Brunnick. 2023.

For our analysis, we distinguished the following four general sections of the bridle mark system:

1. **The melon line:** running from the blowhole to the crease of the rostrum.
2. **The eye line:** running from the melon line to the eye.
3. **The eye patch:** surrounding the eye, sometimes with a lighter brow.
4. **The pre cape:** running from the blowhole, backwards to the base of the skull (Figure 2 and Figure 3).

**Figure 4:**
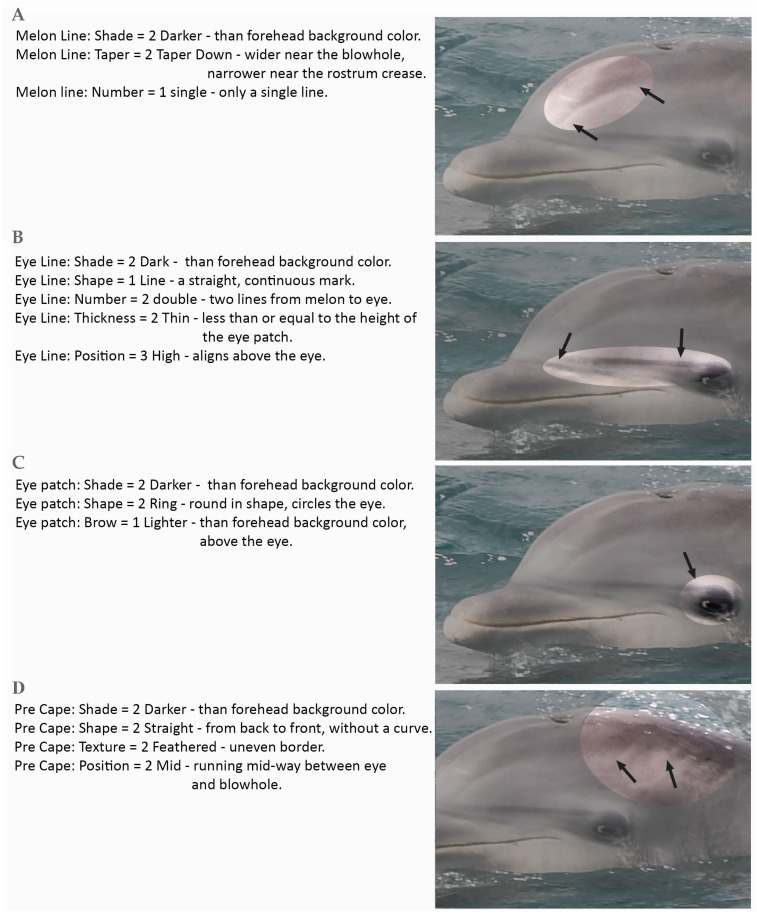
Coding for dolphin #393: Arrows point to area of the areas defined. (**A**) melon line; (**B**) eye line; (**C**) eye patch; (**D**) pre cape. Photographic image collected under NOAA GA# 18152. © Harzen/Brunnick. 2018.

When examining a bridle mark, each of the four sections was coded for the general characteristics of size, shape, shade, and number for a total of 17 codes (Table 1). The shade qualities of hypopigmentation (light) and hyperpigmentation (dark) describe the bridle features compared to the background coloration of the head. Taper up suggests a line was thicker at the bottom, while taper down indicates the line would be thicker at the top. The thickness of the eye line code reflects a comparison to the height of the eye patch, i.e., thin being less than, and thick being more than, the height of the patch (Figure 4).

The authors examined the selected 30 dolphins and independently coded each bridle mark. Once examined, the resulting sequence of numbers for each animal was converted into the 17-digit code assigned to the dolphin based on the descriptive qualifiers in Table 1.

To measure bridle mark distinctiveness, Cohen’s weighted kappa was used to compare numeric patterns based on the 17 bridle mark codes for all possible pairs of dolphins. Pairwise comparisons of N = 30 dolphins generated 435 κ statistics.

Cohen’s kappa coefficient is a statistical metric used to measure inter-observer reliability (agreement between different raters who categorize the same items) and intra-observer reliability (agreement between different ratings by a single rater) for categorical data (Cohen, 1960) [59]. It is more robust than simple percent agreement because it factors out the likelihood of raters agreeing purely by chance.

Kappa is calculated using

κ = (p_o_ − p_e_)/(1 − p_e_)

where p_o_ is the observed percentage of agreement between the raters and p_e_ is the expected percentage of agreement by chance; p_e_ is calculated from the observed data as the probabilities of each observer randomly selecting each category.

Kappa values range from −1 to 1 (κ < 0 worse than chance agreement, κ = 0 equal to chance agreement, and κ = 1 perfect agreement). Interpretations vary by field, but common benchmarks qualify the strength of agreement as slight (κ = 0–0.20), fair (κ = 0.21–0.40), moderate (κ = 0.41–0.60), substantial (κ = 0.61–0.80), and almost perfect (κ = 0.81–1) (Landis & Koch, 1977) [60].

The bridle mark distinctiveness null hypothesis was that bridle patterns were not distinct. This would be supported if most of the comparisons yielded high κ values, indicating strong similarities; thus, the cut-off criterion was substantial to almost perfect agreement (κ = 0.60–1.00). Classic Cohen’s kappa treats categories as strictly unordered nominal categories. Five bridle mark characteristics are measured with unordered dichotomies alternatives (melon mark shade, pre cape shade, pre cape shape, pre cape texture, and rostrum irregularity damage) and two bridle mark characteristics are based on numeric data (melon mark number and eye line number). However, the other 10 of the 17 bridle mark characteristics involve two or more ordered categories. Eight bridle mark characteristics are measured with three levels (melon mark taper, eye line shade, eye line thickness, eye patch shade, eye patch shape, eye patch brow, pre cape position, and rostrum irregularity jaw). Two bridle mark characteristics are measured with four levels (eye line shape and eye line position). When categories have a natural ranking (e.g., eye line position: low, middle, high, or none), a weighted kappa is generally more appropriate (Cohen, 1960) [59]. Kappa considers the matches on the main diagonal. The weighted kappa considers off-diagonal elements as well and was used to evaluate distinctiveness.

## 3. Results

**Assumption 1.** 
*Tursiops truncatus do in fact have bridle marks.*

Bridle marks were found on all 458 dolphins for which we found photos of their head area in our extensive photo database.

**Assumption 2.** 
*Bridle marks for Tursiops truncatus are distinct enough to be useful identifiers.*

Of these 458 dolphins, we drew a subset of 30 (6%) dolphins with photographic records of both dorsal fin and bridle marks spanning at least 7 different years (except one neonate with only three years available) (Mean = 10.00 years, SD 2.94, Range = 7–15 years) to evaluate bridle mark codes that were clearly associated with a dolphin identified with dorsal fin photo-identification. This group consisted of 26 adults of unknown age, 3 calves, and 1 neonate with visible fetal folds.

The authors examined the selected 30 dolphins and coded each bridle mark, using a set of 17 multiple-choice questions. These 30 dolphins were fully coded for bridle mark distinctions of size, shape, shade, and number following the coding schema outlined above in Table 1. The resulting 17-digit code was unique to each dolphin tested, with no two individuals having identical codes (Table 2). Even those with similar codes, including animals sharing 16 of the 17 digits were still visually distinguishable.

**Figure 5:**
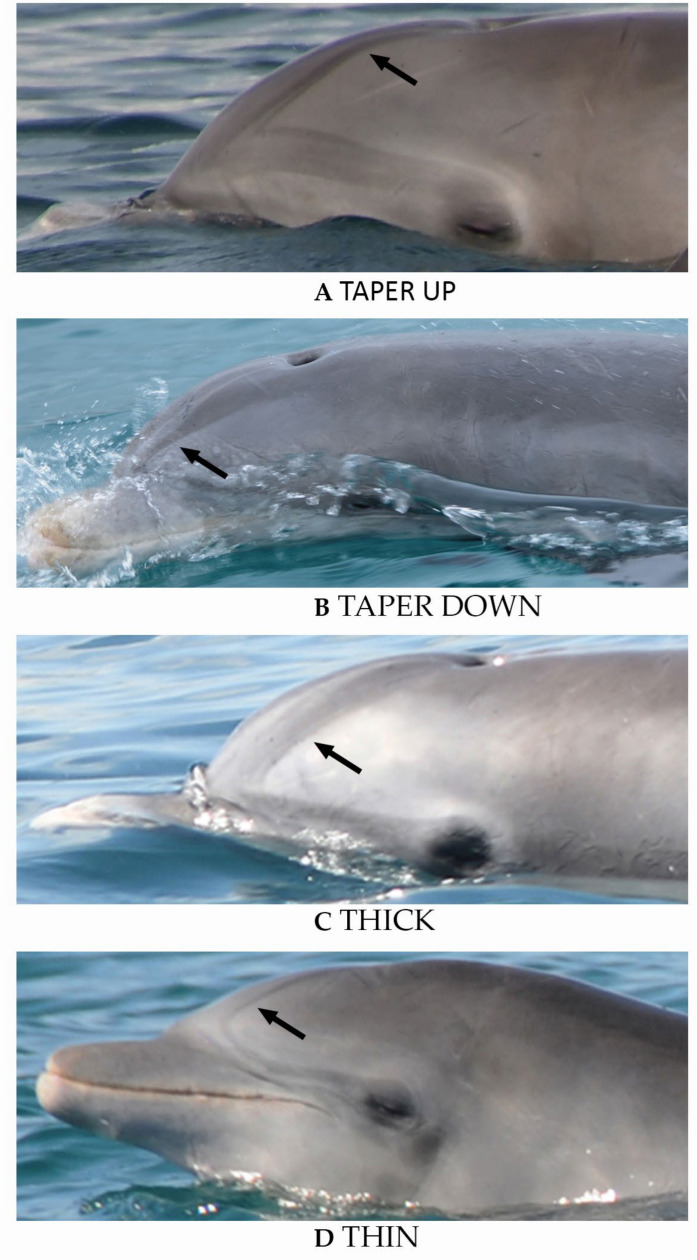
Variety in melon line shape. Arrows point to melon line area described: (**A**) taper up; (**B**) taper down; (**C**) thick; (**D**) thin. Photographic images collected under NOAA GA#s 18152 and 22291. © Harzen/Brunnick. 2023.

**Figure 6:**
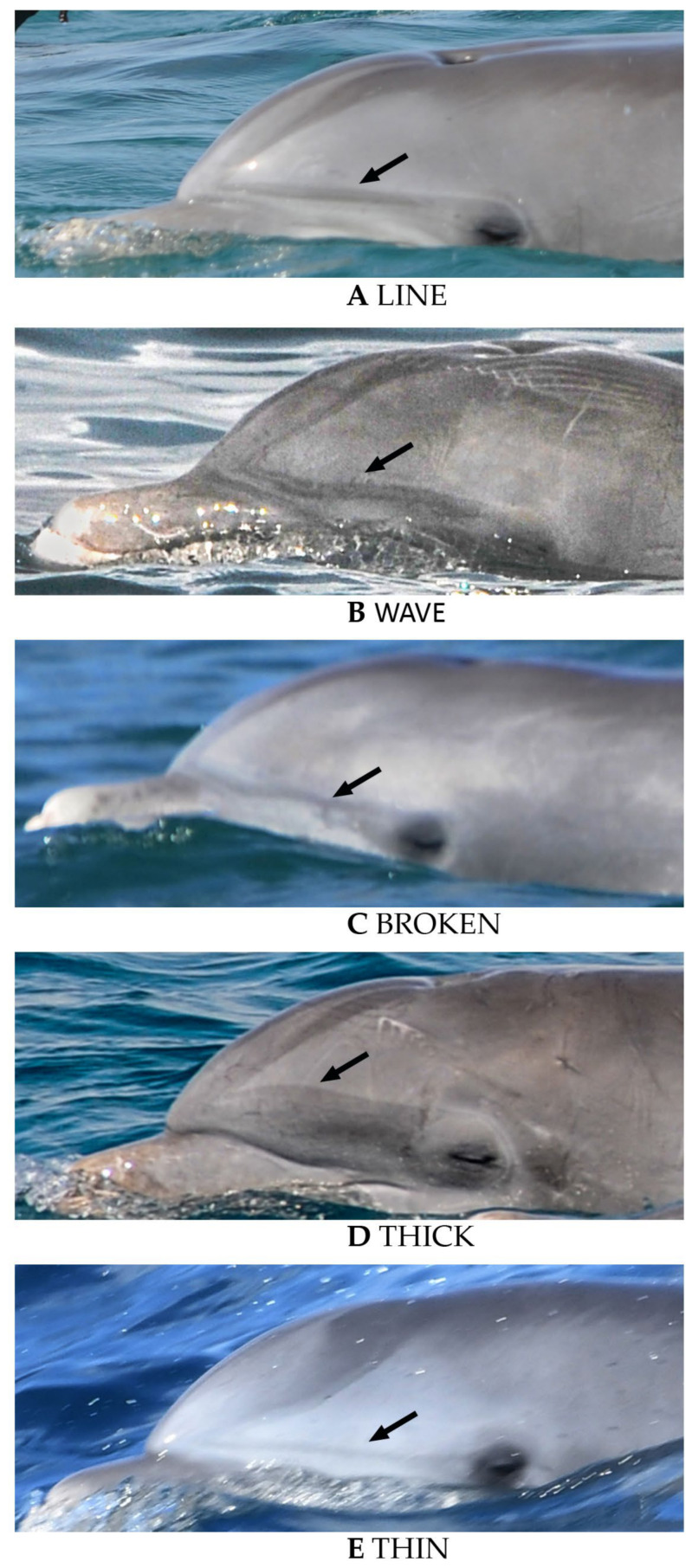
Variety in eye line shape. Arrows point to area of the eye line defined. (**A**) Line—straight; (**B**) wave—curved; (**C**) broken—portion discontinued; (**D**) thick—wide contour; (**E**) thin—narrow contour. Note: in (**B**) wave and (**D**) thick images, white parallel scratches are rake marks from interactions with other dolphins or sharks. Photographic images collected under NOAA GA# 18152. © Harzen/Brunnick. 2015 and 2018.

Comparisons across qualifying codes in Table 2 show that most features of the coded bridle mark system exhibited distinct differences in shape and number. Figure 5 shows examples of the melon line, Figure 6 of the eye line.

The weighted kappa results showed that none of the bridle marks were identical. The majority of the 435 kappa paired comparisons (79%, *n* = 342) had κ values that fit slight, fair, and moderate agreement (slight: *n* = 8, 2%; fair: *n* = 111, 26%, moderate: *n* = 223, 51%). The remaining 21% (*n* = 93 comparisons) had substantial to almost perfect agreement, with most showing substantial (*n* = 90) rather than almost perfect (*n* = 3) agreement. A break-down of the κ substantial category showed that two-thirds of the κ values were 0.60–0.69 (*n* = 67) whereas the other third was composed of *n* = 21 κ values 0.70–0.79 and *n* = 3 κ values 0.80–1.00. These comparisons revealed less distinctive patterns that were nonetheless distinguishable by eye. The null hypothesis that the bridle patterns were not distinct was rejected and the alternative, that the bridle patterns were distinct, was retained.

The three comparisons that suggested almost perfect agreement were visually inspected for the basis of the agreement. Dolphins 93 and 393 (κ = 0.91) had 16 out of 17 identical codes; the exception was eye patch shape. Dolphins 85 and 96 (κ = 0.86) had different codes for two measures (melon mark taper and eye patch shade). Dolphins 96 and 105 (κ = 0.85) also had different codes for two measures (melon mark number and eye line position).

Three comparisons that indicated the greatest distinctiveness were also visually inspected for the basis of bridle mark distinctions. Dolphins 44 and 64 had the most distinct bridle marks (k = 0.11) based on differences in 11 of the 17 bridle measures. Dolphins 47 and 93 were among the most distinct bridle marks (k = 0.12) based on difference in 11 measures. Dolphins 47 and 93 were among the most distinct bridle marks (k = 0.12) based on difference in 10 measures.

**Assumption 3.** 
*Bridle marks for Tursiops remain stable over time*

**Figure 7:**
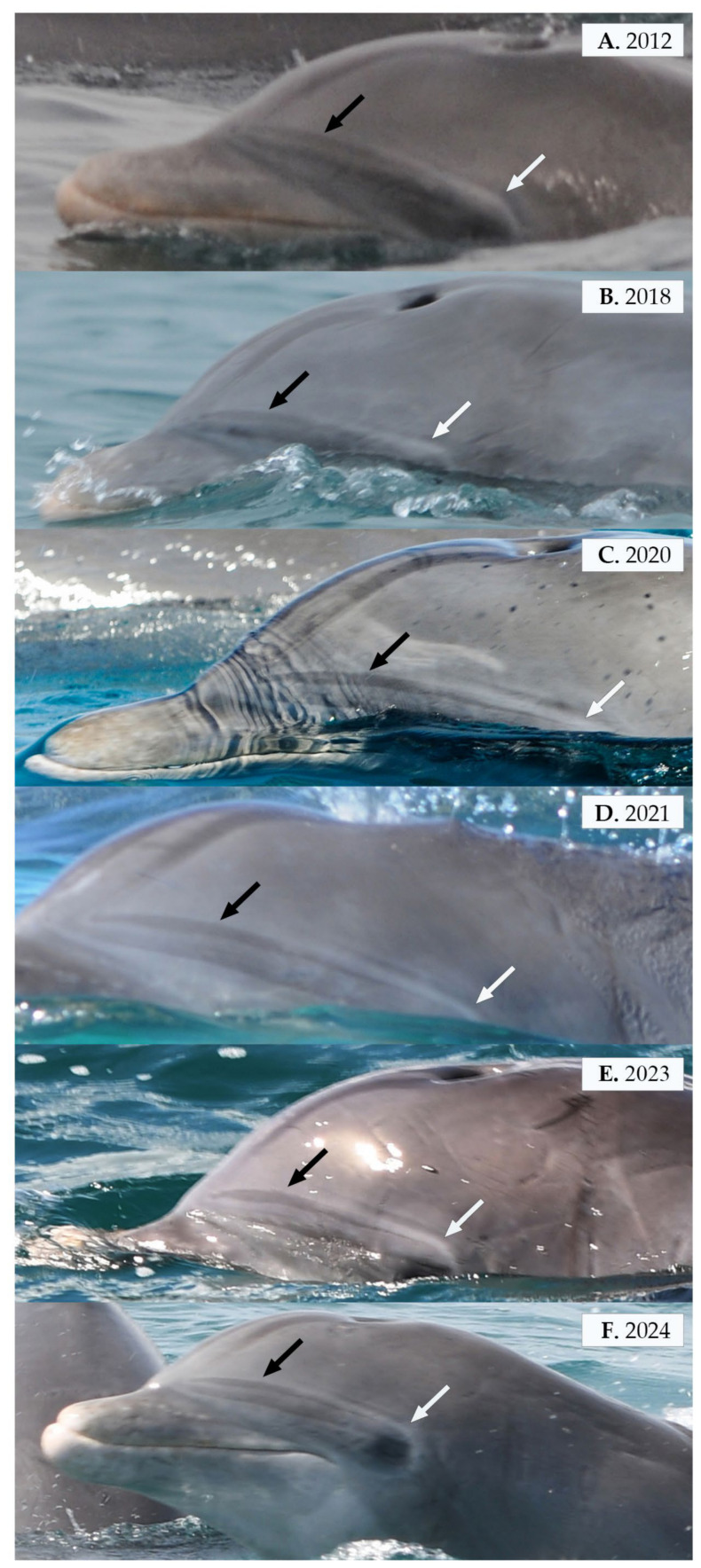
Images of dolphin #185 show that its bridle marks were consistent over a twelve-year period: (**A**) 2012; (**B**) 2018; (**C**) 2020; (**D**) 2021; (**E**) 2023; (**F**) 2024. Black arrows call attention to the dark double eye line. White arrows point to the light-colored brow over and behind the eye. Note: in 2020, light color scratches across the melon line are white rake marks and originate from interaction with another dolphin. Photographic images collected under NOAA GA#s 18152, 13386, and 22291. © Harzen/Brunnick. 2012–2024.

The codes for all individuals listed in Table 2 remained stable over time regardless of the dolphin’s age in the photos or the time between photos. The underlining photo record of the 30 dolphins spanned between 7 and 15 years, except for one neonate whose record only spans 3 years. The oldest and most recent photos of all individuals were independently coded and matched in all cases. While all individuals were adults in their most recent photos, some were calves in the oldest photos. For all individuals the bridle was visible at all ages. For example, dolphin #185 was photographed with the same melon line, and white brow, over a 12-year period (Figure 7).

All dolphins also had shade characteristics, such as melon and eye lines being darker than the background color of the dolphins’ head. This shade distinction is a reported feature for delphinids, resulting from a pre-natal development in color from lighter to darker compared to the background pigmentation, which remains consistent after birth (Perrin, 1997) [52].

Some neonates are born with a darker, blue-black coloration across their entire bodies. This remarkable coloration changes to the more typical gray and white counter-shaded body coloration about 10 days after birth (Cockcroft and Ross, 1990; Mann and Smuts, 1999) [35,61]. Bridle marks remain visible during this transition but are subject to contrast variability.

**Figure 8:**
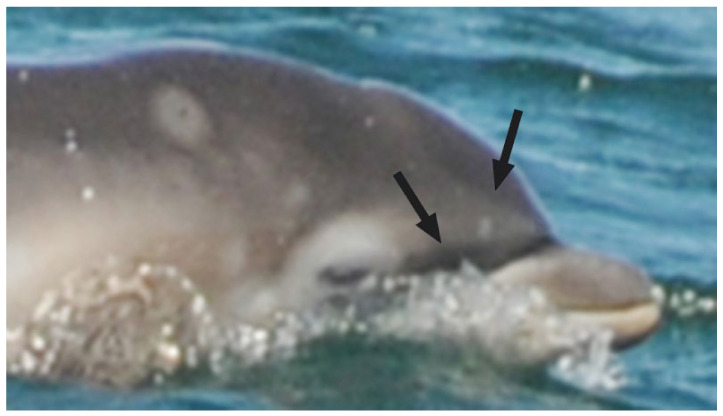
Neonate calf of dolphin #908 with black body coloration. Arrows highlight the eye line and the melon line. Photographic image collected under NOAA GA# 18152. © Harzen/Brunnick. 2017.

**Figure 9:**
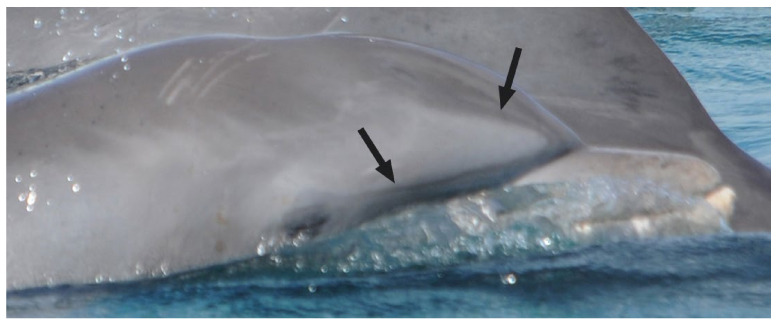
Calf of dolphin #908 one-year later exhibiting the typical dark gray back and lighter gray sides associated with this species. Distinct bridle marks are evident in both images, showing a consistency through a period of maturation designated by a change in overall body color. Note: arrows highlight the eye line and the melon line. Photographic image collected under NOAA GA# 18152. © Harzen/Brunnick. 2018.

For example, the calf of dolphin #908 was observed as a neonate, with dark, blue-black body coloration (Figure 8). Although the contrast between the bridle marks and body color is low, the eye line and the melon line were still discernable. A similar contrast affects the eye patch, which is visibly lighter. Once this calf matured, the eye patch coloration became subtle, while the lighter head coloration makes the melon and eye lines more apparent (Figure 9).

**Figure 10:**
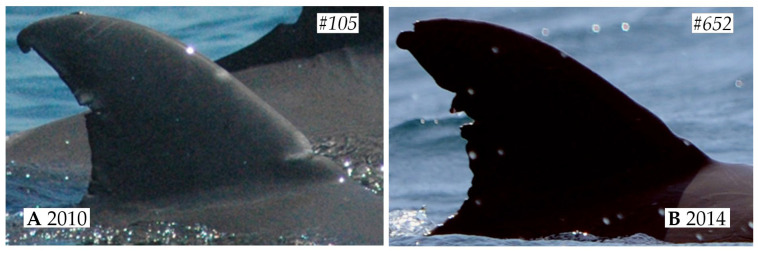
Dorsal fin mark changes: (**A**) dolphin #105 was first added to the catalog in 2010; (**B**) dolphin #652 was added to the catalog in 2014. Photographic images collected under NOAA GA#s 18152 and 22291. © Harzen/Brunnick. 2010 and 2014.

**Figure 11:**
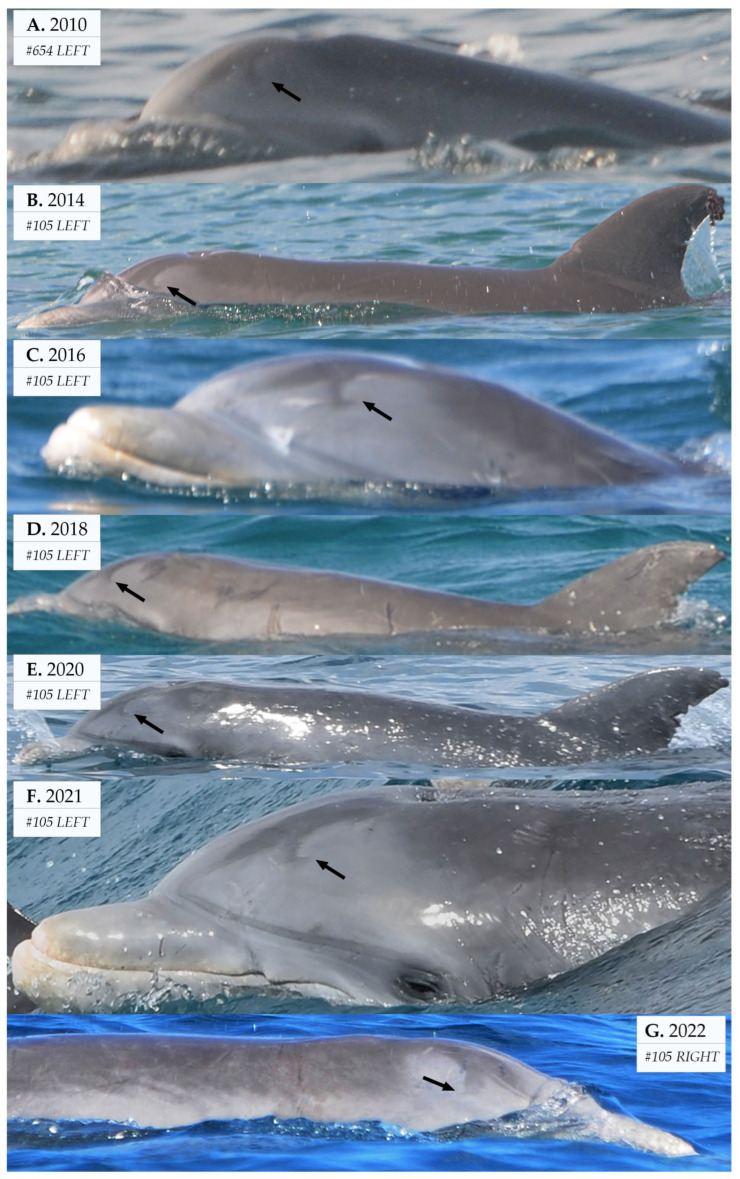
Dolphin #105 and #652 bridle marks over time. Left side (**A**) #105, Melon line for in 2010; (**B**) #652, Melon line in 2014; (**C**) #652 Melon line in 2016; (**D**) #652 Melon line in 2018; (**E**) #652 Melon line in 2020; (**F**) #652, Melon line in 2021. Right Side (**G**) #652, Melon line in 2022. Arrows point to the distinct curve in the melon line. Photographic images collected under NOAA GA#s 18152, 13386, and 22291. © Harzen/Brunnick. 2010–2022.

We also determined that adding bridle marks as a supplement to dorsal fin identification can help prevent errors in the identification process. For example, dolphin #105, which was seen in 2010 and 2011 as an assumed adolescent, displayed some distinct dorsal fin marks, including an indentation at the base of the leading edge (Figure 10A). Dolphin #652, with distinct but different dorsal fin markings than #105, was added to the catalog in 2014 (Figure 10B). During the coding process, it was discovered that the two dolphins had the same bridle mark code and we eventually determined they were in fact the same dolphin (Figure 11), thus eliminating a source of error. Dolphin #105 had experienced subsequent damage that obscured its original identifying dorsal fin marks. Fixing this error not only improved the quality of the dataset but also provided a better understanding of this individual’s life history. There was only one such case among the 30 individuals.

**Figure 12:**
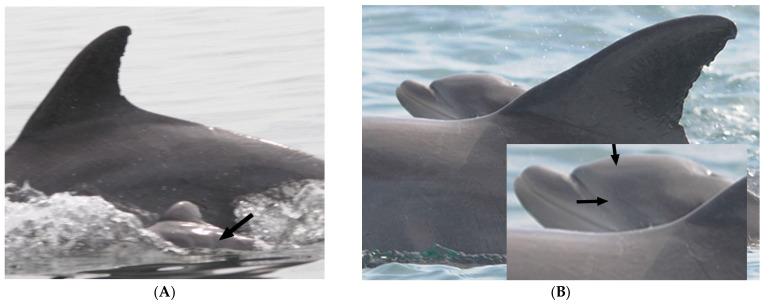
On 14 September 2007, #377, a very small neonate, was observed with #077, the assumed mother: (**A**) arrow highlights fetal folds; (**B**) arrows highlight bridle marks which were subtle due to low contrast in forehead features. Photographic images collected under NOAA GA# 13386. © Harzen/Brunnick. 2007.

Finally, we were able to demonstrate that bridle marks are useful in tracking cow/calf pairs. Clean calves (i.e., those without identifying dorsal fin patterns or scars) are generally identified from any adult swimming in close proximity to the small calf (Grellier et al. 2003) [62]. In one case, a calf, #377, was first seen as a neonate on 17 September 2014. Despite not displaying the darker neonatal coloration described earlier, the bridle mark was visible but suffered from low contrast with the body color, as is common with newborns (Mann and Smuts, 1999) [35] (Figure 12).

**Figure 13:**
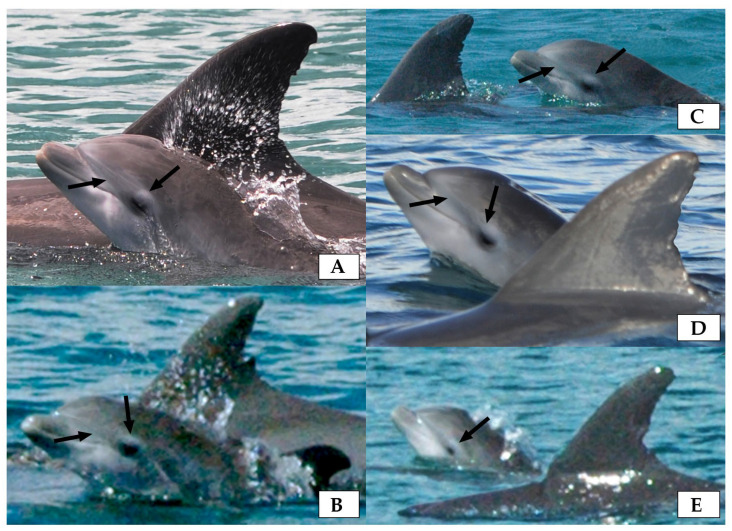
Calf #377 was observed with several adult females on 1 October 2014: (**A**) #377 and #077 (assumed mother); (**B**) #377 and #093 (female); (**C**) #377 and #135 (female); (**D**) #377 and #234 (female); (**E**) #377 and #078 (sibling). Arrows point at the eye line and the eye patch. Photographic images collected under NOAA GA# 13386. © Harzen/Brunnick. 2007.

Two weeks later (1 October 2014), dolphin #377 was photographed swimming near its presumed mother as well as several other adults, three of which are the assumed mothers of older weaned calves that were not seen in this encounter (Figure 13).

**Figure 14:**
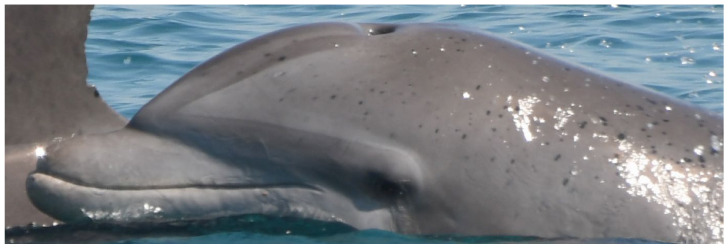
Dolphin #377 in 2020 with bridle mark coloration contrast matured. Photographic image collected under NOAA GA# 22291. © Harzen/Brunnick. 2020.

Although #377’s melon line and eye line were subtle, the enhanced eye patch made the bridle mark useful. If this were the first observation of calf #377, it would have been impossible to distinguish a maternal connection from social behavior. With the addition of bridle mark analysis, we were able to accurately identify this allomaternal/parental connection in this *Tursiops* group (Greenfield et al. 2022; Mann and Smuts, 1998) [37,63]. The bridle mark became more distinct as the dolphin matured and the contrast between marks and background coloration intensified, strengthening the verification by bridle codes (Figure 14).

## 4. Discussion

This study demonstrates that the bridle mark system is an additional method of recognizing individuals within bottlenose dolphin study populations.

Over the past 50 years, irregularities in dolphin dorsal fins (nicks and notches) have been the primary focus for bottlenose dolphin identification (Würsig and Würsig, 1977; Shane, 1977) [14,15]. External forces acting against the dorsal fin frequently result in changes throughout a dolphin’s life, although a certain level of mark stability is required for dorsal fin identification to be exclusive.

Previous research has shown that facial features are suitable as secondary identifiers for many species of delphinids and phocoenids, and in some situations a better first identifier than dorsal fin irregularities, e.g., when dorsal fins are unmarked (Brunnick and Herzing, 1998) [50] or missing entirely, as is the case with the genus *Lissodelphis* (Lipsky et al. 2018) [64]. The bridle mark system is a series of pigmented lines on the forehead, connecting the eye, the rostrum, and the blowhole (Guldberg and Nansen, 1894; Perrin, 1969; Mitchell, 1970; Perrin, 1997; Brunnick and Herzing, 1998; Brunnick, 2000) [50,51,52,53,54,55].

This study confirmed our assumptions that bottlenose dolphins in our study area exhibit the bridle mark system, distinguishable from one individual to the next, which remained stable from neonate through adulthood. The fact that all animals for which photographs of the dolphin head were available display bridle marks suggest that most, if not all, dolphins in the study population have facial features that can be used for identification. Cohen’s kappa coefficient test shows these marks are distinctive in this study, meaning they are a reliable second mark for identification. Due to the extensive testing period in this study, we were able to confidently show bridle systems remain stable and identifiable over periods of up to 12 years, some since infancy.

Therefore, they can be used to identify even young individual dolphins when their dorsal fin markings are less prevalent or absent altogether. Although many young dolphins have similar dorsal fins, our study shows that they are unlikely to have identical bridle marks.

Though often subtler than large notches in some dorsal fins, the longitudinal consistency of bridle marks over years make them very useful when trying to match images taken over many years. As we demonstrated, an adult (dolphin #105, Figure 10) with significant notches in its dorsal fin was matched to an individual previously recorded with less damage to the dorsal fin by comparing melon lines on the forehead. Overall, our study shows that bridle marks combined with dorsal fin identification methodology can reduce identification errors, improve the accuracy of identifying individual dolphins, and as a result help facilitate a more robust understanding of wild dolphin populations.

Bridle marks not only provide a highly reliable way to monitor individual dolphins over time but can also be very helpful for following kinship of wild dolphins. For the calves that do not acquire an identifiable dorsal fin mark prior to weaning, our study has shown that bridle marks can serve as a reliable identifier of individuals independent from observed continued associative conduct, i.e., close contact swimming with another dolphin potentially interpreted as its mother. In addition, using bridle marks to track dolphins from infancy through weaning and into adulthood can provide insight into the survival of dolphins throughout their early development. In cases when a mother dolphin is lost to a study, bridle marks allow for the continued tracking of their calves.

The observed shade characteristics, such as melon lines and eye lines darker than the background color of the dolphins’ head, are a reported feature for delphinids resulting from a pre-natal development in color compared to the background pigmentation, which is consistent after birth (Perrin, 1997) [52]. Some neonates are born with a darker, blue-black coloration across their entire bodies. This remarkable coloration changes to the more typical gray and white counter-shaded body coloration about 10 days after birth (Cockcroft and Ross, 1990; Mann and Smuts, 1999) [35,61]. Bridle marks remain visible during this transition but are often in low contrast with the background color initially, becoming more distinctive with time.

Bridle marks are subject to some of the same quality criteria as dorsal fin photos, including angle, lighting, focus, and quality of the photographs. A most distinctive mark is much more discernable in low-quality photos. The less distinctive the mark, the higher the photo quality is required to be (Bichell et al. 2018; Tyson-Moore et al. 2022) [26,29]. Several photos may be needed to document the entire bridle system, as some photos could show the melon line, while the eye line is still underwater. But once the entire bridle mark system is documented, it is a permanent record and, in many cases, it is not necessary to have subsequent photos of every part of a bridle mark system to make or verify an identification.

The use of bridle marks will not replace dorsal fin identification but is proposed as a secondary, supplemental method allowing for more accurate analysis of existing data. It also provides new opportunities for data collection. For example, traditional photo-identification requires dorsal fin photos to be taken from a perpendicular 90° angle; bridle marks can be seen from several angles, including from the front or above, i.e., aerial imagery. Consequently, drone images capturing bridle marks from above at a straight angle may become a useful addition to future standard image-capturing methodologies (Cheney et al. 2022) [65].

Much like identifying dorsal fin irregularities, such as tear patterns, bridle marks are widely variable across different dolphins. Some bridle patterns are strikingly distinct, while others are more subtle. Just as researchers are required to attain the skills of dorsal fin photo-identification to best identify individual dolphins, practice is also needed to hone the skill of using bridle marks (Urian et al. 2015; Tyson-Moore et al. 2022) [21,26].

Future expansion of this study should include testing of the coding consistency by individuals with different levels of experience. Previous studies that tested the role of proficiency found that matching facial features was significantly different to what would be expected from random matching (Genov et al. 2017; Spencer, 2018) [56,57].

When working with a large number of animals, the coding scheme may assist in matching against sizable datasets by grouping animals with similar traits. This process may reduce the number of possible matches to a manageable level in the manner of FinScan, DARWIN, or the algorithm used to identify leatherback turtles based on spotting (Urian and Wells, 1996; Buonantony, 2008; Melancon et al. 2011; Tyson-Moore et al. 2022) [25,26,66,67]. Given the fast rise of AI models, the 17-point coding system could possibly be developed into an algorithm to help identify individual dolphins from photos of faces in a timely manner, further facilitating the analysis of large datasets.

Additional studies of bridle marks will also be helpful in determining the level of occurrence and variability between *Tursiops* populations as well as other delphinids. Some species may have bridle marks that are difficult to discern due to natural body coloration; therefore, this secondary mark may not benefit all delphinid studies equally.

## 5. Conclusions

The static uniqueness of the bridle mark system on individual dolphins allows for more accurate identification by using a two-mark system. Their use as a second identifier helps overcome dataset gaps in long-term studies of social structure. In studies with consistent and frequent observations, physical changes can be documented and accounted for with relative ease. However, in studies with large data collection intervals, i.e., long gaps between sightings, or low re-sighting rates (open ocean studies, for example) where individual dolphins can be encountered only a few times in a year but continually over multiple years, tracking changes to the dorsal fin can be more difficult. Following calves past weaning when they have achieved a level of independence from their mothers is also challenging. In these cases, bridle marks can provide confidence in individual identification, and we recommend their use whenever possible. Moreover, like computer-aided dorsal fin identification, the 17-point coding system of bridle marks may lend itself to development into an AI algorithm that could help identify individual dolphins from photos of faces in a timely manner.

## Figures and Tables

**Table 1:** The 17-digit coding key for *Tursiops* bridle marks.

Bridle Marks	Codes			
Melon Line: Shade	Light (1)	Dark (2)		
Melon Line: Taper	Taper Up (1)	Taper Down (2)	Thick (3)	None (4)
Melon Line: Number	(1)	(2)		
Eye Line: Shade	Light (1)	Dark (2)	None (0)	
Eye Line: Shape	Line (1)	Wave (2)	Broken (3)	None (0)
Eye Line: Number	(1)	(2)	(3)	None (0)
Eye Line: Thickness	Thick (1)	Thin (2)	None (0)	
Eye Line: Position	Low (1)	Middle (2)	High (3)	None (0)
Eye Patch: Shade	Light (1)	Dark (2)	None (0)	
Eye Patch: Shape	Patch (1)	Ring (2)	None (0)	
Eye Patch: Brow	Light (1)	Dark (2)	None (0)	
Pre Cape: Shade	Light (1)	Dark (2)		
Pre Cape: Shape	Swoop (1)	Straight (2)		
Pre Cape: Texture	Smooth (1)	Feathered (2)		
Pre Cape: Position	High (1)	Mid (2)	Low (3)	
Rostrum Irregularity: Jaw	Underbite (1)	Overbite (2)	None (0)	
Rostrum Irregularity: Damage	Yes (1)	No (0)		

**Table 2:** Thirty individual bridle mark systems coded for size, shape, color, and number [MM = melon mark].

Bridle Mark Characteristics	Dolphin ID#				
	4	5	44	47	60
Melon Line: Shade	2	2	2	2	2
Melon Line: Taper	3	4	2	4	4
Melon Line: Number	1	1	1	1	1
Eye Line: Shade	2	2	2	2	2
Eye Line: Shape	1	1	1	2	1
Eye Line: Number	1	1	1	3	1
Eye Line: Thickness	1	2	1	1	1
Eye Line: Position	2	2	3	1	3
Eye Patch: Shade	1	2	2	1	2
Eye Patch: Shape	2	1	1	2	2
Eye Patch: Brow	0	0	0	0	1
Pre Cape: Shade	2	2	2	2	2
Pre Cape: Shape	2	2	1	1	2
Pre Cape: Texture	1	1	2	2	2
Pre Cape: Position	1	1	2	2	1
Rostrum Irregularity: Jaw	1	0	1	0	1
Rostrum Irregularity: Damage	0	0	0	1	0
Distinguishing Feature	Small hook		Deep hang Pre Cape	White lips	
**Bridle Mark Characteristics**	**Dolphin ID#**				
	**62**	**64**	**70**	**77**	**78**
Melon Line: Shade	2	2	2	2	2
Melon Line: Taper	2	2	2	3	2
Melon Line: Number	1	1	1	2	1
Eye Line: Shade	2	2	2	2	2
Eye Line: Shape	2	2	2	1	1
Eye Line: Number	2	2	3	3	2
Eye Line: Thickness	1	2	1	1	2
Eye Line: Position	3	2	2	3	2
Eye Patch: Shade	2	1	2	2	2
Eye Patch: Shape	1	2	1	1	1
Eye Patch: Brow	0	1	1	1	0
Pre Cape: Shade	2	2	2	2	2
Pre Cape: Shape	1	2	1	2	1
Pre Cape: Texture	1	1	2	1	2
Pre Cape: Position	2	2	2	3	2
Rostrum Irregularity: Jaw	1	0	1	1	0
Rostrum Irregularity: Damage	1	1	0	0	0
Distinguishing Feature	White lips	White lips		Asymmetrical Melon Line	
**Bridle Mark Characteristics**	**Dolphin ID#**				
	**81**	**83**	**85**	**92**	**93**
Melon Line: Shade	2	2	2	2	2
Melon Line: Taper	4	4	4	4	2
Melon Line: Number	1	1	1	1	1
Eye Line: Shade	2	2	2	2	2
Eye Line: Shape	1	3	1	3	1
Eye Line: Number	2	2	2	3	2
Eye Line: Thickness	1	2	2	1	2
Eye Line: Position	2	3	2	2	3
Eye Patch: Shade	1	2	1	1	2
Eye Patch: Shape	2	1	1	2	1
Eye Patch: Brow	1	0	1	1	1
Pre Cape: Shade	2	2	2	2	2
Pre Cape: Shape	2	2	2	1	2
Pre Cape: Texture	2	1	2	2	2
Pre Cape: Position	1	1	2	3	2
Rostrum Irregularity: Jaw	1	1	0	0	1
Rostrum Irregularity: Damage	0	0	0	0	0
Distinguishing Feature	Y scar behind left eye		Thick wave high on Melon Line		
**Bridle Mark Characteristics**	**Dolphin ID#**				
	**96**	**100**	**105**	**112**	**114**
Melon Line: Shade	2	2	2	2	2
Melon Line: Taper	3	3	3	4	4
Melon Line: Number	1	1	2	2	1
Eye Line: Shade	2	2	2	2	2
Eye Line: Shape	1	1	1	2	2
Eye Line: Number	2	2	2	2	3
Eye Line: Thickness	2	1	2	1	1
Eye Line: Position	2	3	3	3	2
Eye Patch: Shade	2	1	2	2	0
Eye Patch: Shape	1	2	1	1	0
Eye Patch: Brow	1	1	1	0	0
Pre Cape: Shade	2	2	2	2	2
Pre Cape: Shape	2	1	2	2	2
Pre Cape: Texture	2	2	2	2	2
Pre Cape: Position	2	3	2	1	1
Rostrum Irregularity: Jaw	0	0	0	0	0
Rostrum Irregularity: Damage	0	0	0	0	0
Distinguishing Feature			Downward hook on Melon Line		
**Bridle Mark Characteristics**	**Dolphin ID#**				
	**135**	**196**	**197**	**233**	**234**
Melon Line: Shade	2	2	2	2	2
Melon Line: Taper	3	3	3	2	4
Melon Line: Number	1	1	1	1	2
Eye Line: Shade	2	2	2	2	2
Eye Line: Shape	1	1	2	3	2
Eye Line: Number	1	1	1	2	2
Eye Line: Thickness	2	1	1	1	1
Eye Line: Position	3	2	2	2	3
Eye Patch: Shade	1	2	1	1	1
Eye Patch: Shape	2	1	2	1	2
Eye Patch: Brow	0	1	1	0	0
Pre Cape: Shade	2	2	2	2	2
Pre Cape: Shape	2	2	2	1	1
Pre Cape: Texture	2	1	2	2	2
Pre Cape: Position	1	3	2	2	3
Rostrum Irregularity: Jaw	1	0	0	0	1
Rostrum Irregularity: Damage	0	0	0	0	0
Distinguishing Feature	Disruption in Melon Line				
**Bridle Mark Characteristics**	**Dolphin ID#**				
	**236**	**377**	**381**	**393**	**394**
Melon Line: Shade	2	2	2	2	2
Melon Line: Taper	3	2	2	2	3
Melon Line: Number	1	1	1	1	2
Eye Line: Shade	2	2	2	2	2
Eye Line: Shape	2	1	2	1	1
Eye Line: Number	3	3	2	2	2
Eye Line: Thickness	1	2	2	2	1
Eye Line: Position	2	2	1	3	2
Eye Patch: Shade	1	1	2	2	1
Eye Patch: Shape	2	2	1	2	2
Eye Patch: Brow	0	0	1	1	1
Pre Cape: Shade	2	2	2	2	2
Pre Cape: Shape	2	2	2	2	2
Pre Cape: Texture	2	2	1	2	2
Pre Cape: Position	2	3	3	2	2
Rostrum Irregularity: Jaw	0	1	0	1	1
Rostrum Irregularity: Damage	0	0	0	0	0
Distinguishing Feature					Spot on Melon Line

## Data Availability

The datasets presented in this article are not readily available because the data are part of an ongoing study. Requests to access the datasets should be directed to the Taras Oceanographic Foundation at taras@taras.org.
